# TREM-1 governs NLRP3 inflammasome activation of macrophages by firing up glycolysis in acute lung injury

**DOI:** 10.7150/ijbs.77304

**Published:** 2023-01-01

**Authors:** Wen-Jing Zhong, Tian Liu, Hui-Hui Yang, Jia-Xi Duan, Jin-Tong Yang, Xin-Xin Guan, Jian-Bing Xiong, Yan-Feng Zhang, Chen-Yu Zhang, Yong Zhou, Cha-Xiang Guan

**Affiliations:** 1 Department of Physiology, School of Basic Medical Science, Central South University, Changsha, Hunan 410078, China; 2 College of Physiology Education, Chongqing University of Arts and Science, Chongqing 412160, China; 3 Department of Geriatrics, Respiratory Medicine, Xiangya Hospital, Central South University, Changsha, Hunan, 410011, China; 4 Department of Cardiovascular Surgery, Xiangya Hospital, Central South University, Changsha, Hunan, China

**Keywords:** Acute lung injury, TREM-1, glycolysis, NLRP3 inflammasome, HIF-1α, macrophages

## Abstract

The triggering receptor expressed on myeloid cells-1 (TREM-1) is a pro-inflammatory immune receptor potentiating acute lung injury (ALI). However, the mechanism of TREM-1-triggered inflammation response remains poorly understood. Here, we showed that TREM-1 blocking attenuated NOD-, LRR- and pyrin domain-containing 3 (NLRP3) inflammasome activation and glycolysis in LPS-induced ALI mice. Then, we observed that TREM-1 activation enhanced glucose consumption, induced glycolysis, and inhibited oxidative phosphorylation in macrophages. Specifically, inhibition of glycolysis with 2-deoxyglucose diminished NLRP3 inflammasome activation of macrophages triggered by TREM-1. Hypoxia-inducible factor-1α (HIF-1α) is a critical transcriptional regulator of glycolysis. We further found that TREM-1 activation facilitated HIF-1α accumulation and translocation to the nucleus* via* the phosphoinositide 3-kinase (PI3K)/AKT/mammalian target of rapamycin (mTOR) pathway. Inhibiting mTOR or HIF-1α also suppressed TREM-1-induced metabolic reprogramming and NLRP3/caspase-1 activation. Overall, the mTOR/HIF-1α/glycolysis pathway is a novel mechanism underlying TREM-1-governed NLRP3 inflammasome activation. Therapeutic targeting of the mTOR/HIF-1α/glycolysis pathway in TREM-1-activated macrophages could be beneficial for treating or preventing inflammatory diseases, such as ALI.

## Introduction

Acute lung injury (ALI), a progressive and devastating clinical condition, is characterized by progressive alveolar-capillary barrier damage, local inflammatory accumulation, denudation of the alveolar epithelium, and hyaline membrane formation 1. Particularly inflammation plays a crucial role in the pathogenesis of ALI 2. Macrophages comprise the front line of defense against the pathogen in the lung 3, 4. We have reported that the depletion of macrophages mitigates ALI in mice 3. The triggering receptor expressed on myeloid cells-1 (TREM-1) is an activating immune receptor, constitutively expressed on monocytes/macrophages and neutrophils 5. TREM-1, coupled to the adaptor protein 12-kDa DNAX activating protein (DAP12) phosphorylation, activates spleen tyrosine kinase (Syk) and phosphatidylinositol-3 kinase (PI3K), resulting in the production of inflammatory cytokines and chemokines 6. Our previous study found that the expressions of TREM-1 in lipopolysaccharide (LPS)-induced ALI mice lungs and macrophages are significantly increased, and blocking TREM-1 mitigates LPS-induced ALI 7, 8. NOD-, LRR- and pyrin domain-containing 3 (NLRP3) inflammasome is a cytosolic signaling complex comprising a sensor molecule, the adaptor apoptosis-associated speck-like protein containing a CARD (ASC), and the effector protease caspase 1. Once activated, the NLRP3 inflammasome induces pro-caspase-1 self-cleavage and activation, mediating the maturation and secretion of interleukin-1 beta (IL-1β), IL-18, and high-mobility group protein B1 (HMGB1) 9. These bioactive cytokines play a pivotal role in initiating and amplifying the inflammatory processes during ALI. Other investigators and we have identified that the NLRP3 inflammasome is a critical inflammatory orchestrator during ALI. NLRP3 deficiency alleviates pancreatitis-associated ALI 10. We have reported intervention factors such as a COX-2/sEH dual inhibitor, vasoactive intestinal peptide (VIP), and epoxyeicosatrienoic acids could attenuate ALI by inhibiting the NLRP3 inflammasome activation in mice 3, 11, 12. Recently we have reported blocking TREM-1 attenuates NLRP3 inflammasome activation in LPS-induced ALI 8. However, the mechanism by which TREM-1 governs NLPR3 inflammasome activation in ALI remains unclear.

Recently glucose metabolic reprogramming has been thought to be a crucial regulator of the NLRP3 inflammasome activation in macrophages 13. Metabolic reprogramming is first described in cancer cells, also called the Warburg effect, characterized by an increase in aerobic glycolysis and a reduction of oxidative phosphorylation (OXPHOS) 14. LPS-induced glycolysis stimulates IL-1β expression by hypoxia-inducible factor-1α (HIF-1α), hexokinase-II (HK2), and pyruvate dehydrogenase kinase M2 (PKM2) activation. Those three molecules of glycolysis are directly involved in IL-1β secretion and NLRP3 inflammasome activation 15. Our study indicates that the blockade of glycolysis partially inhibits the NLRP3 inflammasome activation in LPS-induced ALI 16. In parallel, the NLRP3 inflammasome activation is correlated with glucose transporter 1 (GLUT1)-dependent glycolysis in postburn 17. Glycolysis-related increase in reactive oxygen species (ROS) level contributes to the NLRP3 inflammasome activation and IL-1β secretion 18. So, understanding the role of TREM-1 in regulating glucose metabolic processes is crucial for deciphering how TREM-1 governs NLRP3 inflammasome activation in ALI.

HIF-1α is known to activate transcriptional targets regulating glucose uptake, glycolysis, and flux 19. Thus, activated HIF-1α increases glucose metabolism through glycolysis but the reduced entry of glucose into the OXPHOS. Such metabolic alteration provides immune cells with increased biomass production, thus fueling inflammation 20. HIF-1α signaling is essential for macrophage-mediated inflammation 21. HIF-1α binds to a site approximately 300 bp upstream of the transcription start site of IL-1β, inducing *Il-1β* mRNA expression 22. Nevertheless, whether HIF-1α-mediated metabolism reprogramming is involved in TREM-1-induced inflammation remains unknown. The ubiquitin-mediated proteolysis rapidly degrades HIF-1α under normoxic conditions 23. Regardless, inhibited degradation and accelerated translation are essential to the activation of HIF-1α 24. Phosphoinositide 3-kinase (PI3K)/AKT/ mammalian target of rapamycin (mTOR) pathway is shown to up-regulate the translational initiation of HIF-1α 25. AKT-mTOR-HIF-1α-mediated aerobic glycolysis is known as a metabolic basis for trained immunity 26. In addition, ROS prevents prolyl hydroxylases (PHDs) from modifying HIF-1α (hydroxylation), inducing its accumulation under both hypoxic and normoxic conditions 27.

Here, we hypothesized that TREM-1 instigated HIF-1α accumulation in PI3K/AKT/mTOR-dependent manner, resulting in macrophages' glucose metabolic reprogramming, which was critical to NLRP3 inflammasome activation. Our work reveals a novel association between metabolism and inflammation in macrophages.

## Materials and methods

### Mice and induction of acute lung injury

The IRB of the school of Basic Medical Science at Central South University (Changsha, China) approved all mouse protocols. Male C57BL/6J mice (22 ± 2 g) were purchased from Hunan SJA Laboratory Animal Co., Ltd (Hunan, China). All mice were individually kept in ventilated, temperature- and humidity-controlled environments (24-26 ℃, 50%-60%) under a 12:12 h light: dark cycle. For the ALI model, C57BL/6J mice were intratracheally injected with LPS (5 mg/kg in 50 μL of saline, from* E. coli* O111:B4, Sigma-Aldrich, MO, USA) for 6 h. Control mice were treated with a single intratracheal injection of 50 μL saline. Antagonistic TREM-1 peptide (LR12, LQEEDAGEYGCV, 5 mg/kg) 28 or sequence-scrambled control peptide (LRS, YQVGELCTGEED, 5 mg/kg) was intravenously injected 2 h before the LPS administration. The dodecapeptide was chemically synthesized (GL Biochem, China) as COOH terminally amidated peptides with >95% purity, as confirmed by mass spectrometry and analytical reverse-phase high-performance liquid chromatography. These peptides were free of endotoxin.

### Lung histology and inflammatory injury score

Six hours after the LPS injection, mice were sacrificed. The left lobe was fixed at 4% neutral buffered formaldehyde solution at 4℃. Multiple sections (4 µm) were sliced for hematoxylin and eosin staining (H&E, Solarbio, China, Beijing). Images were taken with Pannoramic Scan (3Dhistech, Hungary, Budapest). The scoring of histological changes was measured. According to five independent variables, the severity of morphologic criteria was graded from 0 to 4: mixed cell alveolar inflammation, bronchoalveolar hyperplasia, hemorrhage, alveolar lipoproteinosis, and hyaline membranes. The lung injury score was performed by three blinded pathologists.

### Isolation and culture of primary mouse peritoneal macrophages

Primary mouse peritoneal macrophages were obtained from C57BL/6J mice. Individual mice were injected with 3 mL 3% sterile thioglycolate (Sigma-Aldrich) intraperitoneally. Three or four days later, macrophages were elicited. Cells were cultured and plated into 6-well plates (2×10^6^ cells/well) or 12-well plates (1×10^6^ cells/well) in RPMI-1640 (Gibco, Life Technologies, Carlsbad, CA) with 10% heat-inactivated bovine calf serum (BCS, Gibco) at 37 °C for 2 h. After non-adherent cells were washed, the adherent cells were cultured for the following experiments.

### Treatments of cells

To estimate the role of TREM-1 in LPS-activated macrophages, we treated cells with LR12 (25 μg/mL) 30 min before LPS (1 ng/mL) stimulation. To study TREM-1-mediated activation, we pre-coated 6-well plates (2×10^6^ cells/well), 12-well plates (1×10^6^ cells/well), or 24-well plates (0.5×10^6^ cells/well) with agonist anti-TREM-1 mAb (10 μg/mL, Mab1187, R&D Systems, USA) overnight at 37 ℃ and washed twice with PBS. Then purified macrophages were subjected to additional stimuli, including glycolysis inhibitor (2-DG, 5 mM, Sigma-Aldrich), mTOR inhibitor (Rapamycin, 100 nM, Beyotime, Jiangsu, China), PI3K inhibitor (LY294002, 25 μM, Beyotime), HIF-1α inhibitor (PX-478, 25 μM, Medchem Express, USA), scavenger of ROS (NAC, 500 μM, Beyotime), or DRP1 inhibitor (Mdivi-1, 100 nM, Medchem Express). The macrophages were cultured for 6 or 24 h and harvested for gene or protein detection.

### Lactate production

Measurement of lactate concentration was utilized with a lactate Assay kit (Sigma-Aldrich) according to the manufacturer's protocol. The main reaction mixture contains a 2 μL lactate probe, 26 μL sample solution, 26 μL lactate assay buffer, and 2 μL lactate mix. The sample was incubated at room temperature for 30 min, and the absorbance was measured at colorimetric (570 nm)/fluorometric (λ ex=535 nm/λ em=587 nm).

### Glucose consumption assay

The glucose level in the supernatant was quantified utilizing a high-sensitivity glucose assay kit (Sigma-Aldrich) according to the manufacturer's protocol. The main reaction mixture contains 45 μL glucose assay buffer, 1 μL glucose probe, 2 μL glucose enzyme mix, 2 μL glucose substrate mix, and glucose standard. It was mixed well by pipetting and incubated the reaction for 30 min at 37 °C. The fluorescence intensity was measured at (λ ex =535 nm/λ em =587 nm) using a Varioskan Flash (Thermo Fisher Scientific).

### Western blot analysis

Lung tissue homogenate and macrophages were harvested, and proteins were extracted using RIPA buffer (Beyotime) containing protease inhibitors cocktail (Roche, Mannheim, Germany). To concentrate supernatants for western blot, 700 μL 100% methanol and 175 μL trichloromethane were added to 700 μL supernatant and vortexed for 30 s. Supernatants were then centrifuged at 13000 rpm for 5 min at 4°C. The supernatant liquid was removed, and added 700 μL 100% methanol, then centrifuged at 13000 rpm for 5 min at 4°C. Supernatants were discarded. And the remaining pellet was resuspended in 20 μL10% SDS, then added 4 μL 5×SDS-PAGE sample loading buffer (Beyotime) and boiled for 10 min at 95°C. The protein concentrations were measured with Pierce™ Rapid Gold BCA Protein Assay Kit (Thermo Fisher Scientific, Grand Island, USA).

Equal amounts of protein or all protein from supernatants were subjected to 8%-12% gradient polyacrylic amide gel under reducing conditions. Gels were transferred into polyvinylidene difluoride membranes (Millipore, USA), blocked with 5% fat-free milk or 5% albumin from bovine serum (BSA, Biofroxx, Germany) at room temperature for 1.5 h. The blots were reacted with the primary antibody at 4 °C overnight, followed by horseradish peroxidase-conjugated secondary antibody (1:1000; Cell Signaling Technology, USA), and detection by ChemiDoc XRS (Bio-Rad, USA). The intensities of the bands were quantified using the Image Lab Analyzer software (Bio-Rad). β-actin, α-tubulin, or GAPDH were used as a loading control. The antibodies used in the study are shown in Table 1.

### Real-time PCR

Total RNA was isolated from macrophages and lungs using RNAiso (TaKaRa Clontech, Japan). Reverse transcription with approximately 1 μg of total RNA was carried out in a T100^TM^ Thermal Cycler (Bio-Rad, USA) using PrimeScript^TM^ RT reagent Kit (TaKaRa Clontech). Targeted gene expressions were measured by quantitative real-time PCR analyses using SYBR^®^ Premix Ex Taq™ II system (TaKaRa Clontech) on a Bio-Rad real-time PCR system (CFX96 Touch™; Bio-Rad, USA). The qPCR program was initiated at 95 °C for 30 s; 40 cycles of 95 °C for 15 s, and 60 °C for 30 s. β-actin was used as an endogenous reference gene. The primers in Table 2 were purchased from Sangon Biotech (Shanghai, China). Gene expression abundance was calculated by the 2^-ΔΔCt^ method.

### Cytokine detection

Tumor necrosis factor-alpha (TNF-α) and IL-1β contents in the cell culture supernatant were measured after an additional 24 h of incubation using appropriate ELISA kits (Cat# TNF-α: 88-7324; IL-1β: 88-7013; Invitrogen, Thermo Fisher Scientific, USA).

### Immunofluorescence

To image HIF-1α nuclear translocation and mitochondria morphology, macrophages were stimulated with Mab1187 (10 μg/mL) for 24 h and then washed with PBS three times for 5 min, fixed with 4 % paraformaldehyde for 15 min. The cells were incubated with 0.2% Triton X-100 and blocked with 1% BSA for 30 min before being stained with anti-mouse HIF-1α (1:100, Novus) overnight at 4°C. After washing 3 times, the cells were incubated with Alexa Fluor 488-conjugated anti-mouse IgG antibody overnight at 4°C (ABclonal, China). The nuclei were counterstained with DAPI (Solarbio, China). After that, coverslips were mounted with a drop of ProLong Gold antifade mounting reagent (Solarbio). Images were captured with a fluorescence microscope (Nikon, Tokyo, Japan). HIF-1α-positive regions were determined using ImageJ software.

### Evaluation of oxidative stress

Intracellular ROS was measured by a ROS assay (Jiancheng Bioengineering Institute, Nanjing, China) according to the manufacturer's protocol. The main reaction mixture contains dichloro-dihydro-fluorescein diacetate and a 10 μM fluorescent indicator of cytosolic ROS. After incubating the reaction for 30 min in the dark, an immunofluorescence photograph was measured using a fluorescence microscope (Nikon, Japan).

### Statistical analysis

Data represented in this study were repeated at least three times and expressed as the mean ± SD. Normally distributed data were analyzed using a one-way analysis of variance (ANOVA) for comparisons among multiple groups. Comparisons between the two-group were made with an unpaired *t*-test. *P*-value < 0.05 was regarded as statistically significant. Statistical analyses were conducted using SPSS 22.0 (IBM, Chicago, IL) or GraphPad Prism 7.0 (San Diego, CA, USA). N represents experiments performed on individual mice or different macrophages from separate mice.

## Results

### Pharmacologic blockade of TREM-1 attenuated LPS-induced lung injury and glycolysis in mice

**Figure 1:**
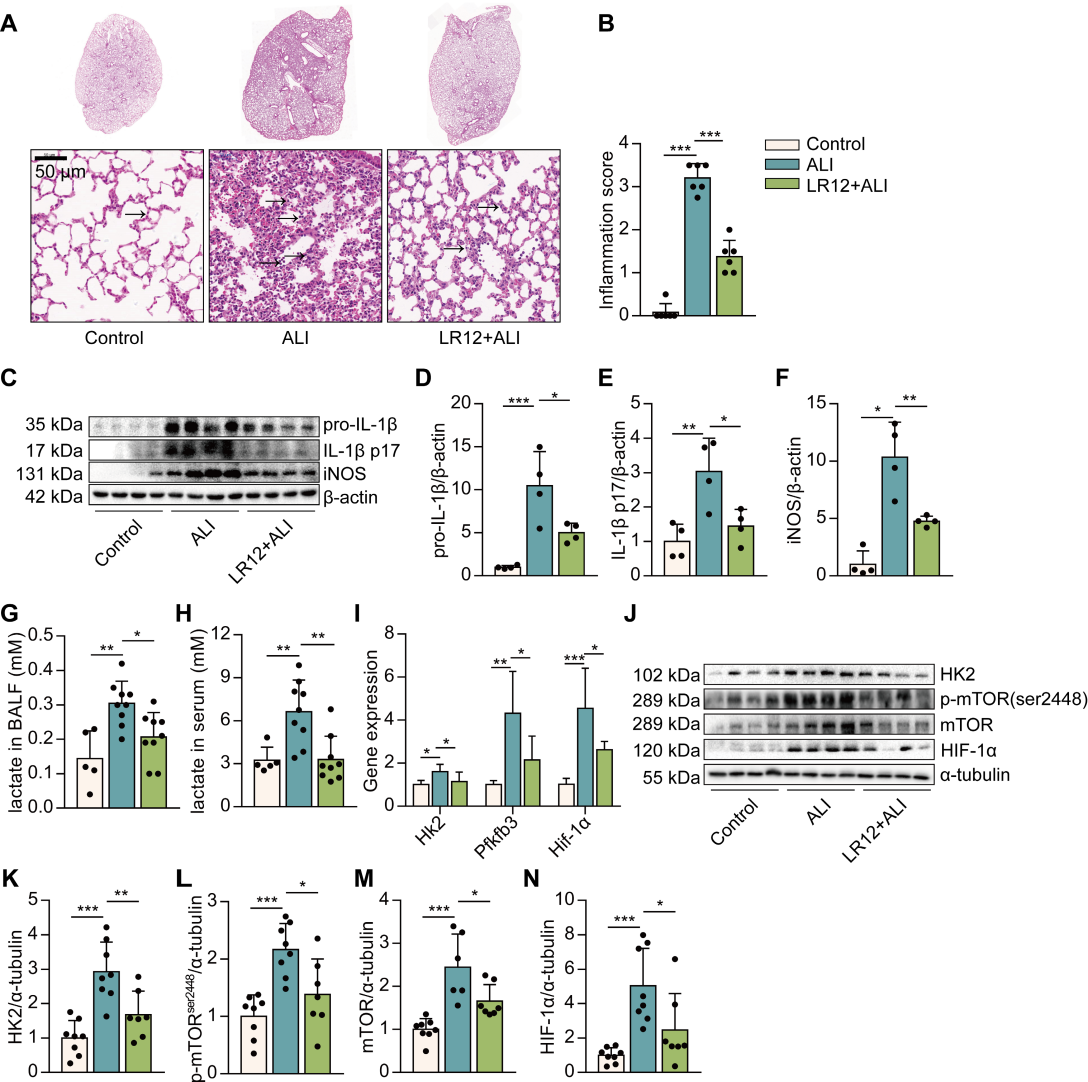
Blockade of TREM-1 reduced intrapulmonary inflammation and limited glycolysis in LPS-induced ALI mice. C57BL/6J mice were intravenously injected with LR12 (5 mg/kg) 2 h before the LPS administration (5 mg/kg, *i.t.*). (A) Six hours later, mouse lungs were excised, and lung histopathology was performed with H&E staining. One representative picture of six mice in each group is shown. (B) Inflammation score was measured, *n*=6 mice/group. (C) Pro-IL-1β, IL-1β p17, and iNOS in the lung lysates were assessed by western blot with β-actin as a loading control. (D-F) The western blot results were quantitated using Image Lab,* n*=8 mice per group. (G-H) Lactate concentration in BALF and serum was assayed, *n*=4-9 mice/group. (I) Expression of *Hk2*, *Pfkfb3,* and *Hif-1α* mRNA in the lungs was detected by real-time PCR. Data was normalized to housekeeping gene β-actin, *n*=5-10 mice/group. (J) Glycolysis-associated proteins of HK2, p-mTOR, mTOR, and HIF-1α in the lung lysates were assessed by western blot with α-tubulin as a loading control. (K-N) Quantification of indicated protein levels in (J), *n*=6-8 mice/group. In all cases, the experiment was repeated twice. Dots represent individual animal values. Statistical analysis was performed using One-way ANOVA adjusted by Tukey's multiple comparison test for Control vs. ALI or ALI vs. LR12+ALI. Error bars indicate mean ± SD. * *P* < 0.05, ** *P* < 0.01, and *** *P* < 0.001. Original western blots represented in graphs are available in Figure 1-source data.

It has been reported that blocking TREM-1 partially inhibits NLRP3 inflammasome activation in LPS-induced ALI, and glycolysis is a crucial regulation of NLRP3 inflammasome activation in macrophages. So, we would like to investigate whether blocking TREM-1 glucose metabolism would alter ALI. We first exposed mice to LR12, a TREM-1 decoy receptor, validated in rodents 28. Histological study showed that LR12-treated mice lungs had less leukocyte infiltration, alveolar congestion, and alveolar barrier thickening than LPS-treated lungs (**Figure 1A-B**). The levels of IL-1β, IL-1β p17, and nitric oxide synthase (iNOS) in the lungs were significantly decreased in LR12-treated mice (**Figure 1C-F**). Then we analyzed changes in gluconic metabolism. We found that the levels of lactate, the end production of aerobic glycolysis, in BALF and serum were similarly decreased in LR12-treated mice (**Figure 1G-H**). LR12 treatment significantly attenuated the expression of genes encoding enzymes in the glycolytic pathway, including *Hk2*, fructose-2,6-biphosphatase 3 (*Pfkfb3*), and *Hif-1α* (**Figure 1I**). HK2 catalyzes the first step in glucose metabolism. The levels of HK2 in the lungs were decreased in LR12-treated mice (**Figure 1J-K**). mTOR activation is sufficient to stimulate glycolysis 29. LR12 treatment significantly reduced the LPS-induced phosphorylated (p)-S2448-mTOR and mTOR levels in the lungs (**Figure 1J, L, M**). The level of HIF-1α in the lungs was significantly decreased in LR12-treated mice (**Figure 1J, N**). Together, these data illustrate that TREM-1 inhibition attenuates intrapulmonary inflammation and limits glycolysis.

### TREM-1 activation induced glucose metabolic reprogramming in macrophages

**Figure 2:**
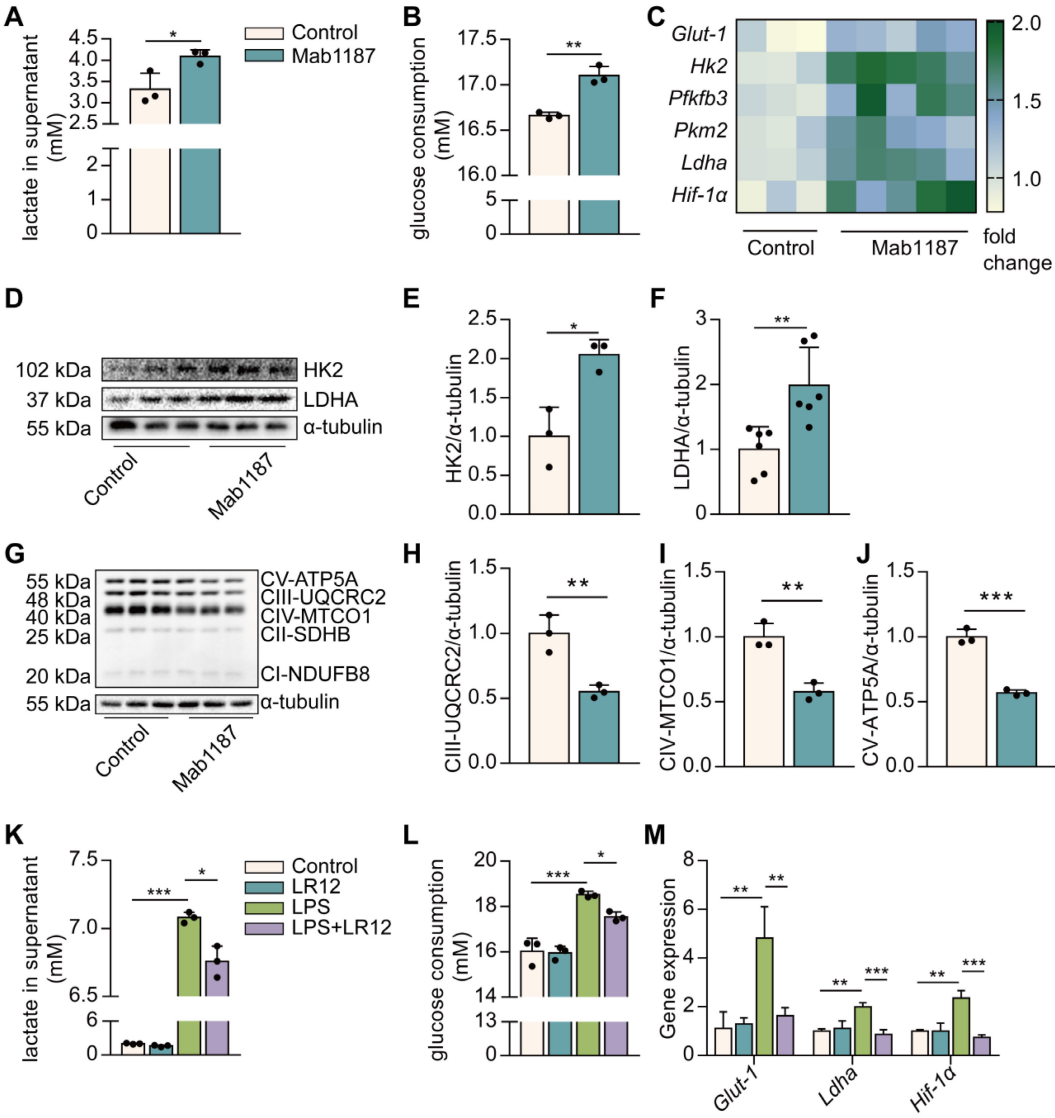
TREM-1 activation instigated glucose metabolic reprogramming in macrophages. Macrophages were stimulated with 10 μg/mL of an agonist anti-TREM-1 mAb. **(A)** Six hours later, the lactate in the supernatant was assayed, *n*=3. **(B)** Glucose consumption in the supernatant in control or 24 h TREM-1-activated macrophages, *n*=3. **(C)** *Glut-1*, *Hk2*, *Pfkfb3*, *Pkm2*,* Ldha,* and *Hif-1α* mRNA in control or 6 h TREM-1-activated macrophages. Data was normalized to housekeeping gene β-actin, *n*=3-5. **(D-F)** Western blot and quantification of the glycolytic enzymes (HK2 and LDHA) in the control or 24 h TREM-1-activated macrophages, *n*=3. **(G)** OXPHOS-related proteins: ATP5A, MTCO1, UQCRC2, SDHB, and NDUFB8, in the macrophages were detected by western blot. **(H-J)** Quantification of indicated protein levels in (G), *n*=3. Macrophages were administrated with LR12 (25 μg/mL) 30 min before the LPS stimulation (1 ng/mL). Twenty-four hours later, **(K)** the lactate and **(L)** relative glucose consumption in the supernatant were assayed, *n*=3. **(M)** Expression of *Glut-1*, *Ldha,* and *Hif-1α* mRNA in macrophages was detected by real-time PCR, *n*=3. *n* represents experiments performed on different macrophages from separate mice. Bar graphs represent mean ± SD. Student's *t*-test (two-tailed, unpaired) was used to compare Mab1187 and Control in (A-J): * *P* < 0.05, ** *P* < 0.01, and *** *P* < 0.001. One-way ANOVA adjusted by Tukey's multiple comparison test was used in (K-M): * *P* < 0.05, ** *P* < 0.01, and *** *P* < 0.001.

To systematically profile metabolic alterations in TREM-1-activated macrophages, we observed lactate production, glucose consumption, and carbohydrate metabolic enzymes. The agonist anti-TREM-1 Ab (Mab1187) has been shown to activate TREM-1 30. Notably, TREM-1 activation increased lactate production and glucose consumption (**Figure 2A, B**). GLUT1 plays an essential role in glucose uptake in macrophages 31. We showed that TREM-1 activation up-regulated the *Glut1* gene expression on macrophages (**Figure 2C**). In addition, TREM-1 activation was accompanied by increased expression of key glycolytic enzymes, including *Hk2*, *Pfkfb3*, *Pkm2*, lactate dehydrogenase A (*Ldha*), and glycolysis critical transcription factor *HIF-1α* in macrophages (**Figure 2C**). Protein expression of HK2 and LDHA was also increased in TREM-1-activated macrophages (**Figure 2D-F**). We further observed that TREM-1 activation was accompanied by a decrease in mitochondrial complex III, IV, and V protein levels (**Figure 2G-J**). These TREM-1-activated macrophages exhibited a skewed profile, favoring glycolytic factors over oxidative regulators. In addition, coincubation with LR12 partially restored metabolic alteration induced by LPS in macrophages. LPS-mediated increase in lactate production, glucose consumption, and glycolysis genes, such as *Glut-1*, *Ldha*, and *Hif-1α* mRNA expression, was significantly diminished by LR12 (**Figure 2K-M**). Collectively, these data illustrate that TREM-1-activated macrophages become more glycolytic but less OXPHOS.

### TREM-1 activation triggered the NLRP3 inflammasome activation

**Figure 3:**
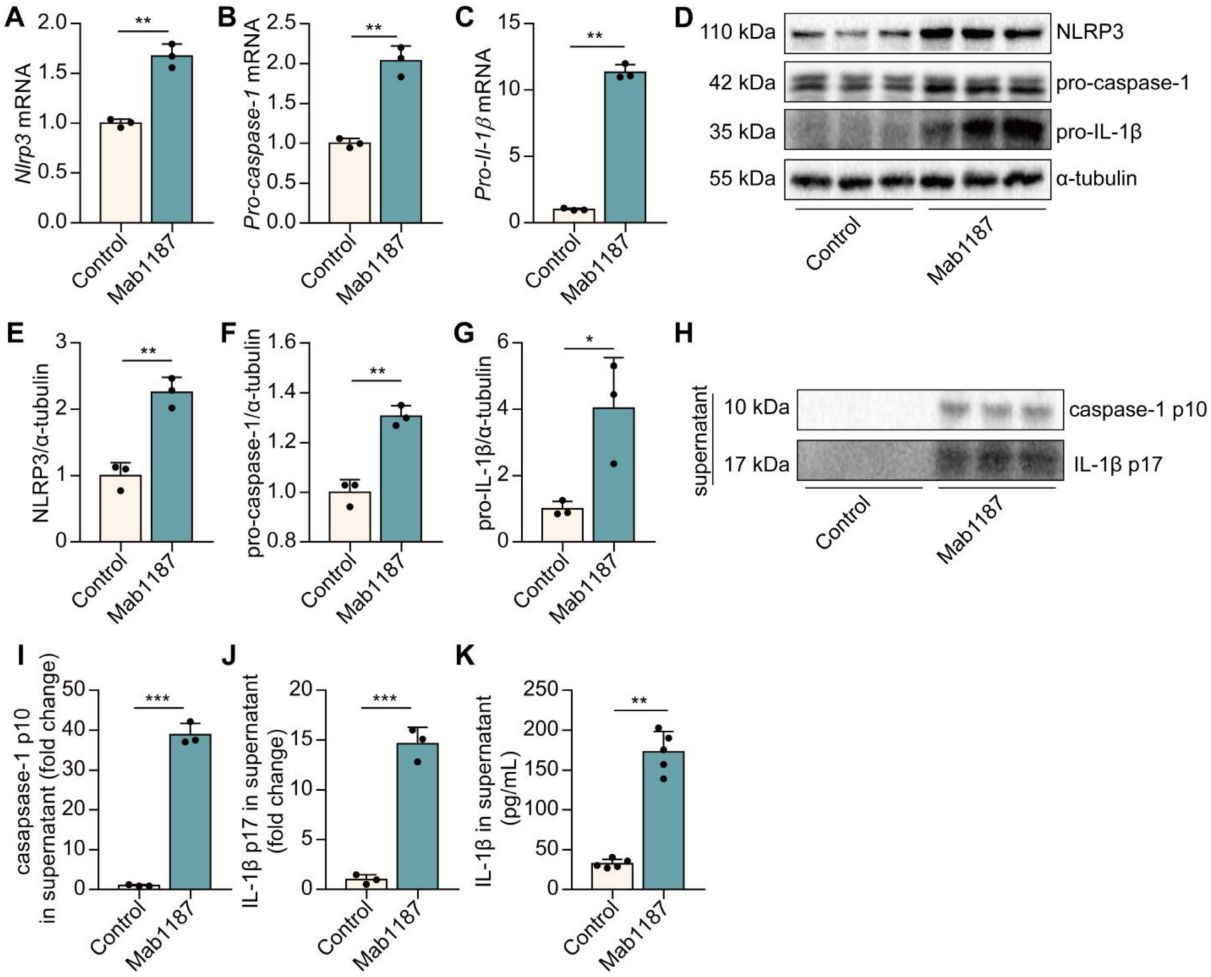
TREM-1 triggered the NLRP3 inflammasome activation in macrophages. Macrophages were incubated with plate-bound isotype-matched control or plate-bound anti-TREM-1 mAb (10 μg/mL). **(A-C)** Six hours later, *Nlrp3*, *Pro-caspase-1,* and *Pro-il-1β* mRNA expressions in macrophages were measured using qPCR. Data was normalized to housekeeping gene β-actin, *n*=3. **(D)** Twenty-four hours later, protein expression of NLRP3, pro-caspase-1, and pro-IL-1β in the cell lysate was detected by western blot with α-tubulin as a loading control. **(E-G)** Quantification of indicated protein levels in (D), *n*=3. **(H-J)** Caspase-1 p10 and IL-1β p17 in the supernatant were detected by western blot, *n*=3. **(K)** IL-1β contents in the supernatants were analyzed with ELISA, *n*=5. *n* represents experiments performed on different macrophages from separate mice. Bar graphs represent mean ± SD. Student's *t*-test (two-tailed, unpaired) was used to compare Control and Mab1187: * *P* < 0.05, ** *P* < 0.01, and *** *P* < 0.001.

We have found blockade of TREM-1 partially inhibited the NLRP3 inflammasome activation 8. Here, we examined the direct effect of TREM-1 agonism on driving the NLRP3 inflammasome. The NLRP3 inflammasome activation requires two steps: priming and activation. NLRP3 complex formation is a crucial step for the priming of NLRP3 inflammasome 32. First, we found that activating TREM-1 with an immobilized agonistic mAb (Mab1187) increased the mRNA level of *Nlrp3*, *Pro‐caspase‐1,* and *Pro‐il‐1β* in macrophages (**Figure 3A-C**). LR12 treatment decreased *Nlrp3*, *Pro‐caspase‐1*, and *Pro‐il‐1β* mRNA in LPS-induced macrophages (**Supplementary Figure 1A-C**). Besides, Mab1187 also strongly increased the protein expressions of NLRP3, pro-caspase-1, and pro-IL-1β (**Figure 3D-G**). These results illustrate that TREM-1 activation promotes NLRP3 inflammasome priming. Caspase-1 p10 and caspase-1 p10 cleave pro-IL-1β into IL-1β p17, biomarkers for NLRP3 inflammasome activation 33. We found that caspase-1 p10 and IL-1β p17 secretion were markedly induced by TREM-1 activation in the supernatant (**Figure 3H-J**). Besides, the secretion of IL‐1β was up-regulated in the supernatant of TREM-1-activated macrophages (**Figure 3K**). Altogether, these data indicate that TREM-1 has direct activating effects on the NLRP3 inflammasome.

### Blockade of glycolysis reversed TREM-1-mediated NLRP3 inflammasome activation in macrophages

**Figure 4:**
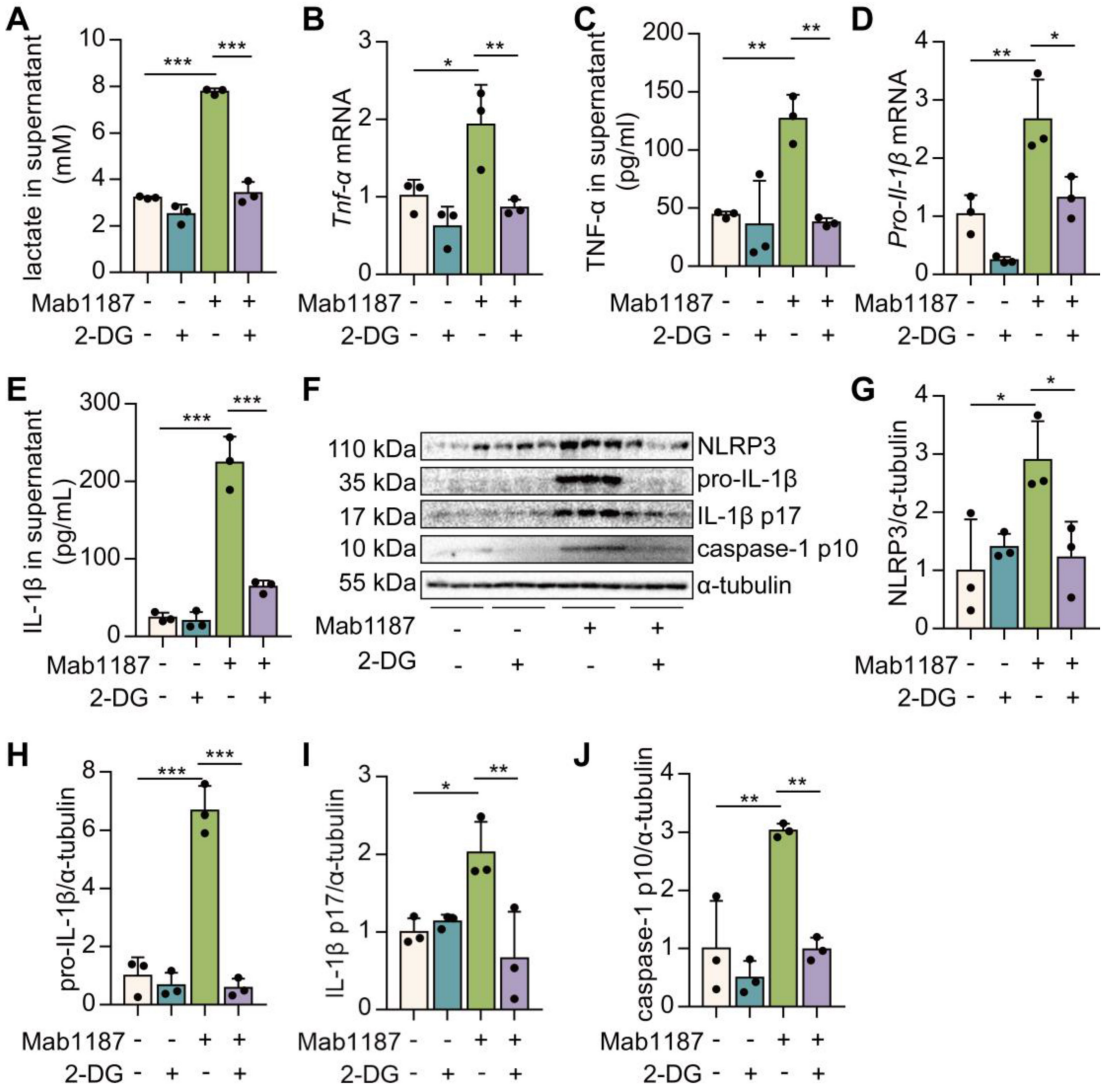
Blockade of glycolysis partially inhibited TREM-1-mediated NLRP3 inflammasome activation in macrophages. **(A)** Macrophages (1×10^6^ cells/well) were premixed with PBS control or 2-DG (5 mM) and then plated into 12-well plates with agonist anti-TREM-1 mAb (10 μg/mL). After 24 h, supernatants were analyzed for lactate production, *n*=3. **(B, D)** Macrophages were treated as in (A), and 6 h later, *Tnf‐α* and *Pro-il-1β* mRNA levels in macrophages were measured using qPCR, *n*=3. **(C, E)** Macrophages were treated as in (A). Twenty-four hours later, TNF‐α and IL-1β production in the supernatant was measured by ELISA, *n*=3. **(F)** NLRP3, pro-IL-1β, IL-1β p17, and caspase-1 p10 protein in cell lysate were detected by western blot, *n*=3. **(G-J)** Quantification of indicated protein levels in (F). *n*=3 biological replicates. Data are expressed as the mean ± SD. One-way ANOVA adjusted by Tukey's multiple comparison test was used. * *P* < 0.05, ** *P* < 0.01, and *** *P* < 0.001.

To assess the role of glycolysis in TREM-1-mediated NLRP3 inflammasome activation, we used an inhibitor of glycolysis, 2‐deoxyglucose (2‐DG). First, 2-DG suppressed the lactate production induced by TREM-1 activation (**Figure 4A**), indicating the pretreatment of 2‐DG inhibited metabolic reprogramming triggered by TREM-1 activation. Then, we demonstrated that TNF-α production after 2-DG administration was significantly decreased in TREM-1-activated macrophages (**Figure 4B-C**). We observed that 2-DG reduced the expression of NLRP3 in macrophages treated with an agonist anti-TREM-1 Ab (**Figure 4F-G**). 2-DG inhibition decreased *Il-1β* gene expression and pro-IL-1β synthesis induced by TREM-1 activation in macrophages (**Figure 4D, F, H**). These results suggest that glycolysis plays a crucial role in the TREM-1-induced NLRP3 inflammasome priming signal. Besides, 2-DG also reduced IL‐1β secretion induced by TREM-1 activation (**Figure 4E**). Protein levels of caspase-1 p10 and IL-1β p17 induced by TREM-1 activation were decreased by 2-DG treatment (**Figure 4F, I, J**), implying that glycolysis plays a vital role in TREM-1-induced NLRP3 inflammasome activation. Furthermore, we found that inhibition of glycolysis using 2-DG decreased TREM-1 expression in the lung of LPS-induced ALI (**Supplementary Figure 2A-B**) or LPS-induced macrophages (**Supplementary Figure 2C**). Collectively, these results suggest that glycolysis is necessary for TREM-1-induced NLRP3 inflammasome activation.

### Functional HIF-1α expression was induced by TREM-1 activation in macrophages

**Figure 5:**
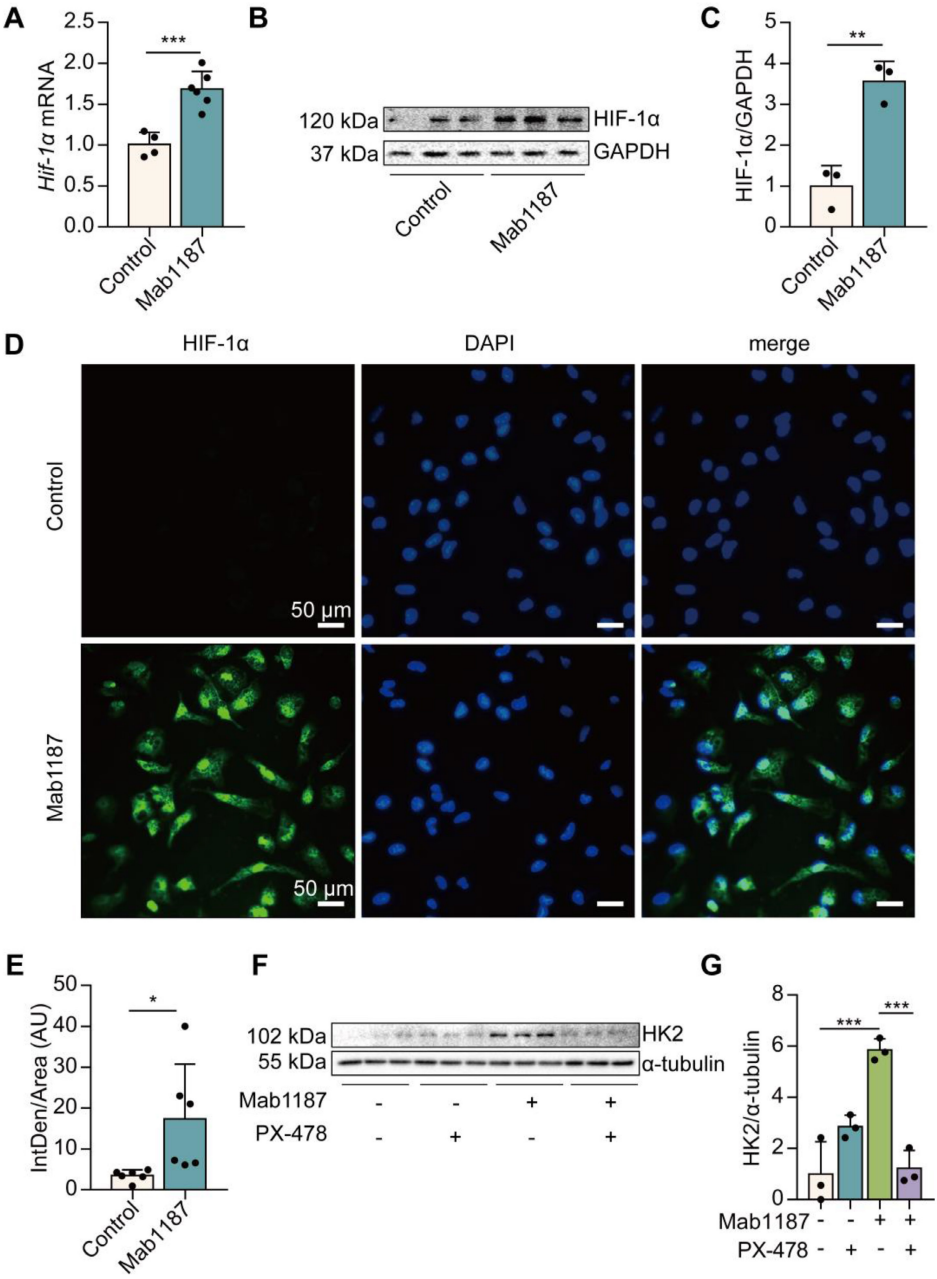
TREM-1 activation induced HIF-1α accumulation and translocation to the nucleus in macrophages. Macrophages were incubated with anti-TREM-1 mAb (10 μg/mL) in a normoxia condition. **(A)** Six hours later, *Hif‐1α* gene levels were measured using qPCR, *n*=4-6. **(B-C)** Twenty-four hours later, the protein of HIF‐1α was performed by western blot with GAPDH as a loading control, *n*=3. **(D)** Macrophages were cultured on anti-TREM-1 for 24 h and then subjected to immunofluorescence examination to analyze the HIF-1α accumulation and translocation to the nucleus (scale bar, 50 μm). **(E)** Average fluorescent intensity was calculated by HIF-1α^+^ fluorescence intensity (IntDen)/area of the region (Area) using ImageJ, *n*=6. **(F-G)** 1×10^6^ cells/well were premixed with PBS control or PX-478 (25 μM) for 30 min, then plated into 12-well plates with agonist anti-TREM-1 mAb (10 μg/mL). HK2 protein levels were measured after an additional incubation for 24 h, *n*=3. *n* represents experiments performed on different macrophages from separate mice. Data are expressed as the mean ± SD. Student's *t*-test (two-tailed, unpaired) was used to compare Mab1187 and Control in (A-E): * *P* < 0.05, ** *P* < 0.01, and *** *P* < 0.001. One-way ANOVA adjusted by Tukey's multiple comparison test was used in G: *** *P* < 0.001.

We next investigated how TREM-1 activation induced glycolysis in macrophages. HIF-1α mediates metabolic reprogramming towards a glycolytic phenotype by inducing the expression of glycolytic enzymes, such as Glut-1, HK2, PKM2, and LDH 34. We found that TREM-1-activated-macrophages expressed significantly higher levels of HIF-1α mRNA and protein, even under normoxic conditions (**Figure 5A-C**). Furthermore, as a functional consequence, HIF-1α accumulation and translocation to the nucleus were also significantly increased in the TREM-1 activation group through immunofluorescence (**Figure 5D-E**). Macrophages treated with PX-478, a HIF-1α inhibitor, significantly inhibited HIF-1α accumulation and translocation to the nucleus induced by TREM-1 activation (**Supplementary Figure 3A**). PX-478 treatment displayed a decreased protein level of HK2, a HIF-1α target gene, in response to TREM-1 activation (**Figures 5F-G**). These results indicate that TREM-1 activation promotes HIF-1α stabilization in macrophages even under normoxic conditions.

### HIF-1α-mediated glycolysis governed the NLRP3 inflammasome activation induced by TREM-1 in macrophages

**Figure 6:**
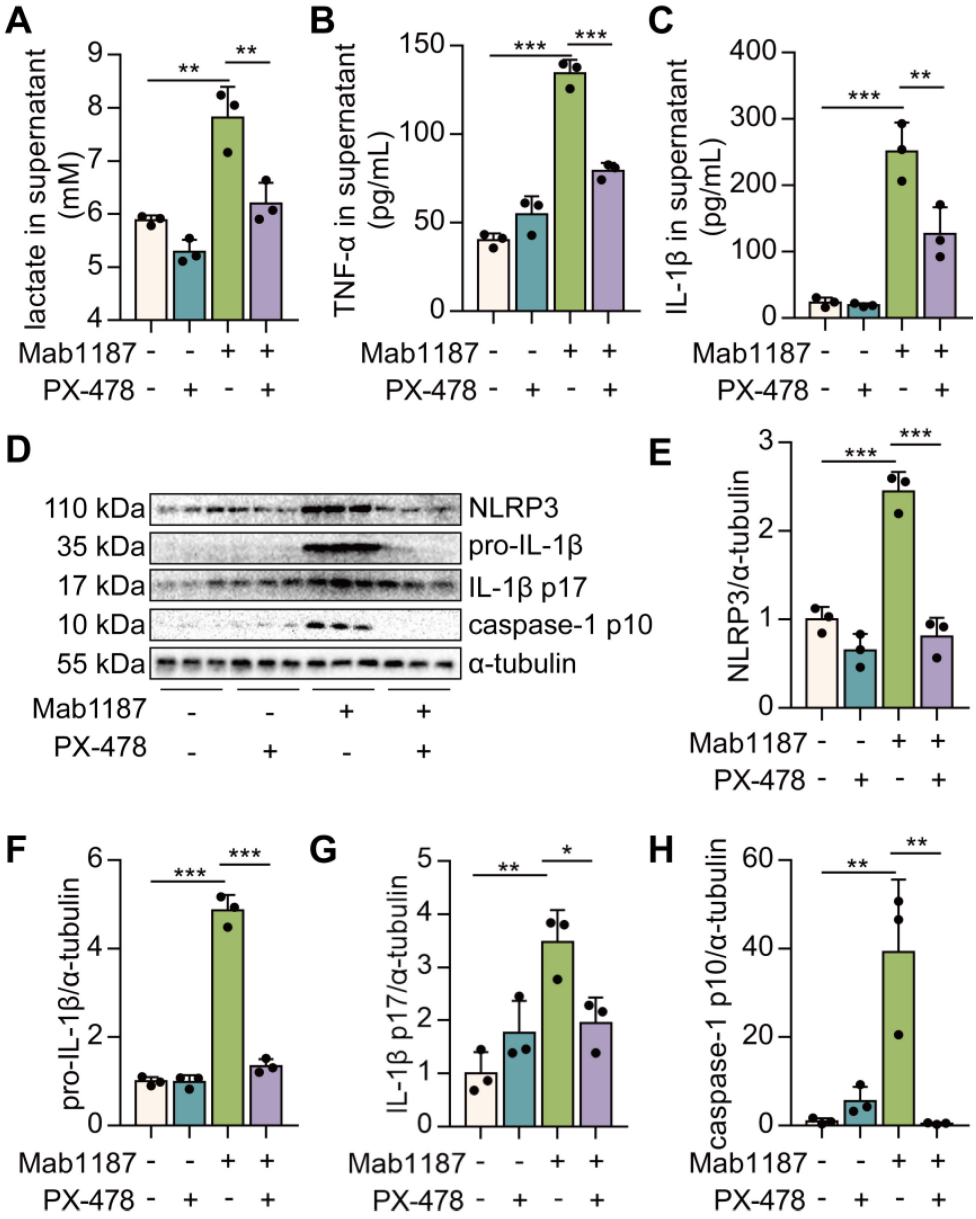
HIF-1α droves TREM-1-mediated NLRP3 inflammasome activation. Macrophages were premixed with PBS control or PX-478 (25 μM) before incubating with plate-bound agonistic anti-TREM-1 mAb (10 μg/mL). **(A)** Twenty-four hours later, lactate level in supernatant was assayed, *n*=3. **(B-C)** The concentration of TNF‐α and IL-1β in the supernatant was assayed using ELISA, *n*=3. **(D)** Protein expression of NLRP3, pro-IL-1β, IL-1β p17, and caspase-1 p10 in macrophage lysate was detected by western blot. **(E-H)** Quantification of indicated protein levels in (D), *n*=3 biological replicates. Statistical analysis was performed using One-way ANOVA adjusted by Tukey's multiple comparison test. Data are expressed as the mean ± SD. * *P* < 0.05, ** *P* < 0.01, and *** *P* < 0.001.

HIF-1α is reportedly a critical transcriptional regulator of immunity and inflammation 21. We treated macrophages with PX-478 to assess the role of HIF-1α in TREM-1-induced NLRP3 inflammasome activation. First, inhibition of HIF-1α suppressed the lactate production induced by TREM-1 activation (**Figure 6A**), suggesting inhibition of HIF-1α blocked the glycolysis. Then, we found that TNF-α production induced by TREM-1 activation was reduced after PX-478 administration in macrophages (**Figure 6B**). PX-478 reduced the expression of NLRP3 and pro‐IL‐1β in TREM-1-activated macrophages (**Figure 6D-F**). Besides, PX-478 also decreased IL‐1β secretion induced by TREM-1 activation (**Figure 6C**). Protein levels of caspase-1 p10 and IL-1β p17 were also reduced by HIF-1α inhibition in TREM-1-activated macrophages (**Figure 6D, G, H**). Altogether, these results indicate the critical role of the HIF-1α signal in TREM-1-mediated NLRP3 inflammasome activation in macrophages.

### TREM-1 activation promoted HIF-1α accumulation by activating PI3K/AKT/mTOR signaling

**Figure 7:**
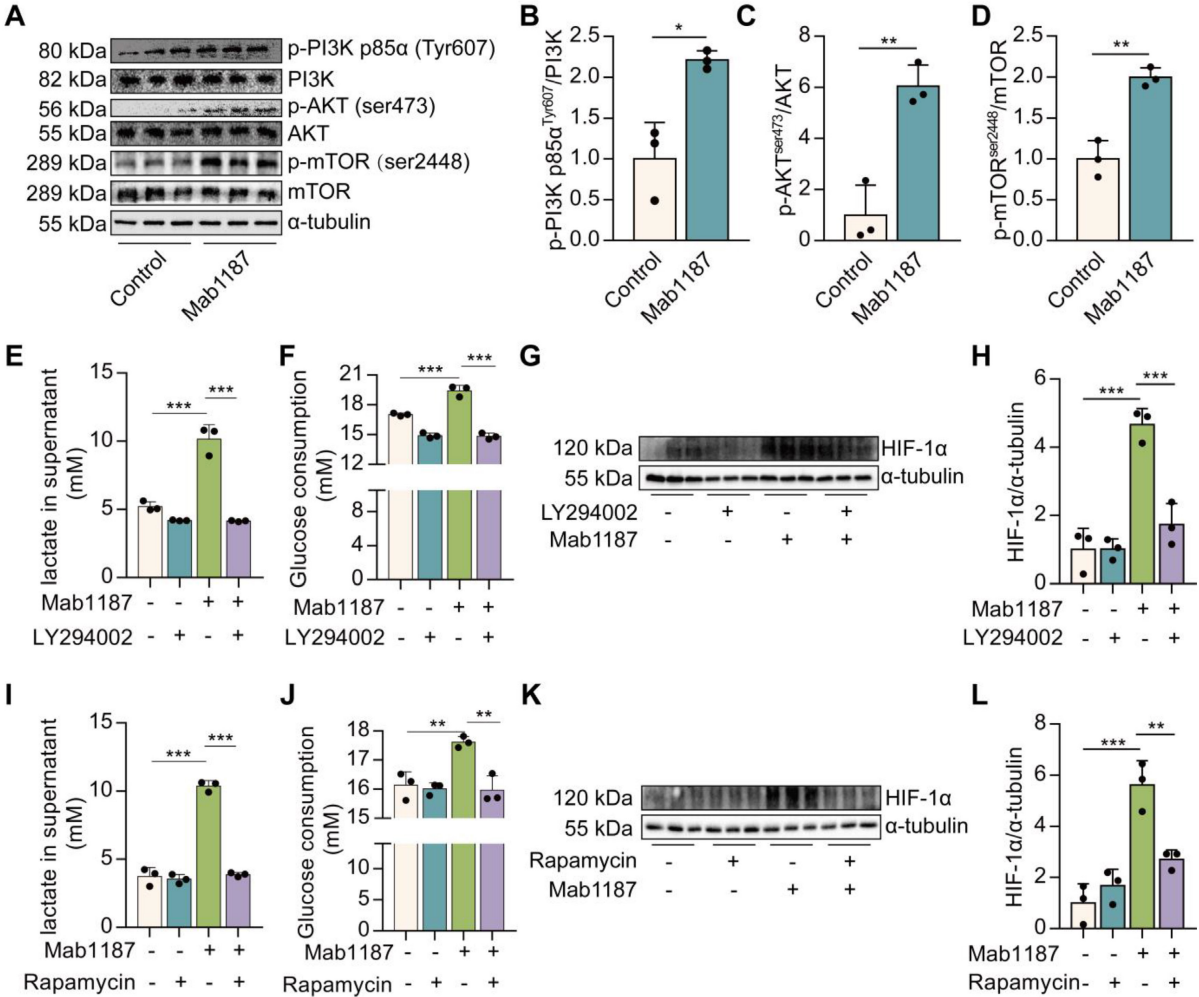
TREM-1 activation stimulated HIF-1α accumulation *via* PI3K/AKT/mTOR signaling. 1×10^6^ macrophages/well were plated into 12-well plates with agonist anti-TREM-1 mAb (10 μg/mL). **(A)** p-PI3K p85α^T607^, total PI3K, p-AKT^s473^, AKT, p-mTOR^s2448^, and mTOR protein levels in control or 24 h TREM-1-activated macrophages. **(B-D)** Quantification of p-PI3K p85α^T607^, p-AKT^s473^ and p-mTOR^s2448^ in (A), *n*=3 biological replicates. Statistical analysis was performed using Student's *t*-test (two-tailed, unpaired). **(E)** Lactate secretion, **(F)** glucose consumption in the supernatant, and **(G-H)** HIF-1α protein levels in control or 24 h TREM-1-activated macrophages co-treated with or without LY294002 (25 μM). *n*=3 biological replicates. Statistical analysis was performed using One-way ANOVA. **(I)** Lactate secretion, **(J)** glucose consumption in the supernatant, and **(K-L)** HIF-1α protein levels in control or 24 h TREM-1-activated macrophages co-treated with or without Rapamycin (100 nM). *n*=3 biological replicates. Statistical analysis was performed using One-way ANOVA adjusted by Tukey's multiple comparison test. Data were expressed as the mean ± SD. * *P* < 0.05, ** *P* < 0.01, and *** *P* < 0.001.

HIF-1α is rapidly degraded under normoxic conditions. The PI3K/AKT/mTOR pathway has been reported stimulating HIF-1α stabilization and transactivation domain function 26. mTOR is a key glucose metabolic regulator 29. PI3K/AKT mediated signaling is upstream of mTOR. We found that the anti-TREM-1 Ab triggered PI3K, AKT, and mTOR phosphorylation in macrophages (**Figure 7A-D**). Inhibition of PI3K (LY294002) or mTOR (Rapamycin) showed a reduction in lactate production and glucose consumption induced by anti-TREM-1 Ab (**Figure 7E-F, 7I-J**), suggesting that PI3K/AKT/mTOR signaling is necessary for TREM-1-driven metabolic reprogramming. Meanwhile, PI3K or mTOR inhibition decreased HIF-1α protein induced by TREM-1 activation (**Figure 7G-H, 7K-L**). These results indicate the requirement for the PI3K/AKT/mTOR signaling pathway in HIF-1α accumulation in response to TREM-1 activation. Moreover, we treated macrophages with Rapamycin and found that Rapamycin decreased TREM-1-induced NLRP3 inflammasome assembly (**Supplementary Figure 4D-F**). Besides, Rapamycin reduced TREM-1 activation-induced IL‐1β secretion, caspase-1 p10, and IL-1β p17 production (**Supplementary Figure 4C, D, G, H**), indicating that TREM-1-mediated NLRP3 inflammasome activation is dependently on mTOR signal in macrophages. In addition, ROS is a potential inducer of HIF-1α by inhibiting its degradation 27. The data showed that TREM-1 activation significantly increased the level of intracellular ROS in macrophages (**Supplementary Figure 5A-B**). However, the scavenger of ROS by N-Acetylcysteine (NAC) failed to reverse the HIF-1α protein expression induced by TREM-1 activation (**Supplementary Figure 5C-D**), suggesting that HIF-1α accumulation induced by TREM-1 is independent on ROS. Collectively, these data suggest that TREM-1 instigates HIF-1α accumulation in PI3K/AKT/mTOR-dependent manner, critical to the NLRP3 inflammasome activation.

### TREM-1 triggered the NLRP3 inflammasome activation by enhancing mitochondrial fission in macrophages

**Figure 8:**
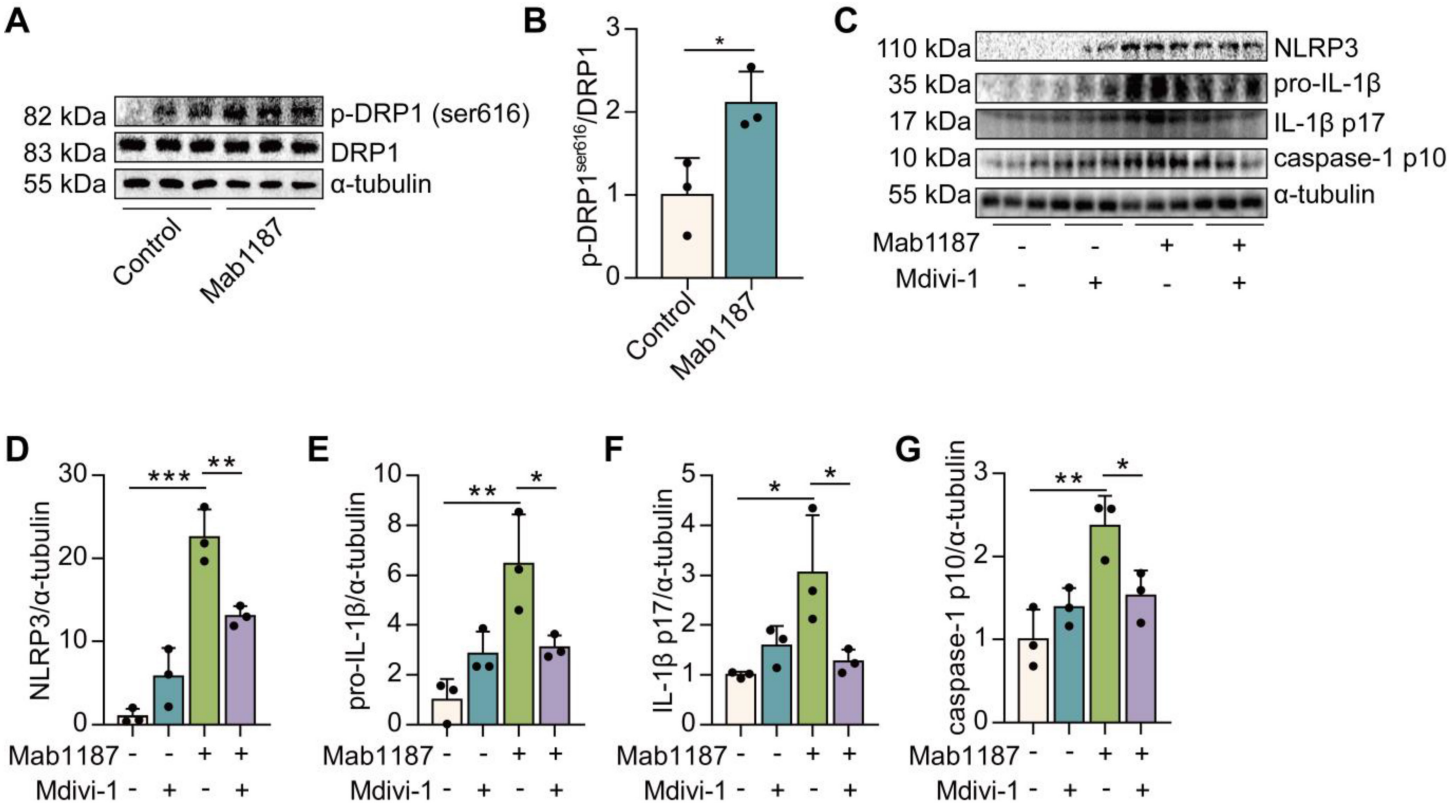
TREM-1 triggered the NLRP3 inflammasome activation by enhancing mitochondrial fission in macrophages. Macrophages were stimulated with 10 μg/mL of an agonist anti-TREM-1 mAb. **(A-B)** Western blot and quantification of p-DRP1^ser616^ and DRP1 in control or 24 h TREM-1-activated macrophages. *n*=3 biological replicates. Statistical analysis was performed using Student's *t*-test (two-tailed, unpaired). Macrophages were premixed with PBS control or Mdivi-1 (100 nM) before incubating with plate-bound agonistic anti-TREM-1 mAb for 24 h. **(C)** Protein levels of NLRP3, pro-IL-1β, IL-1β p17, and caspase-1 p10 in macrophages were detected by western blot. **(D-G)** Quantification of NLRP3, pro-IL-1β, IL-1β p17, and caspase-1 p10 (C), *n*=3 biological replicates. Statistical analysis was performed using One-way ANOVA. Data were expressed as the mean ± SD. * *P* < 0.05, ** *P* < 0.01, and *** *P* < 0.001.

Mitochondrial fission reportedly inhibits OXPHOS by decreasing electron transport chain efficiency 35. Thus, we speculated that TREM-1-inhibited OXPHOS might be involved in mitochondrial fission in macrophages. Mitochondrial fission is mainly mediated by the phosphorylation of dynamin-related protein 1 (DRP1) at serine 616 (S616) 36. We found that DRP1^S616^ phosphorylation was significantly increased by TREM-1 activation (**Figure 8A-B**). Then, we treated macrophages with Mdivi-1, an inhibitor of DRP1, to assess the role of mitochondrial fission in TREM-1-mediated NLRP3 inflammasome activation. We found that Mdivi-1 inhibited the expressions of NLRP3 and pro‐IL‐1β induced by TREM-1 activation (**Figure 8C-E**). Besides, Mdivi-1 reduced TREM-1 activation-induced IL-1β p17 production (**Figure 8C, F**). Protein expression of caspase-1 p10 induced by TREM-1 activation was also decreased by Mdivi-1 in macrophages (**Figure 8C, G**). Altogether, these results indicate that TREM-1 promotes mitochondrial fission through DRP1^S616^ phosphorylation, which inhibits OXPHOS and triggers NLRP3 inflammasome activation.

## Discussion

**Figure 9:**
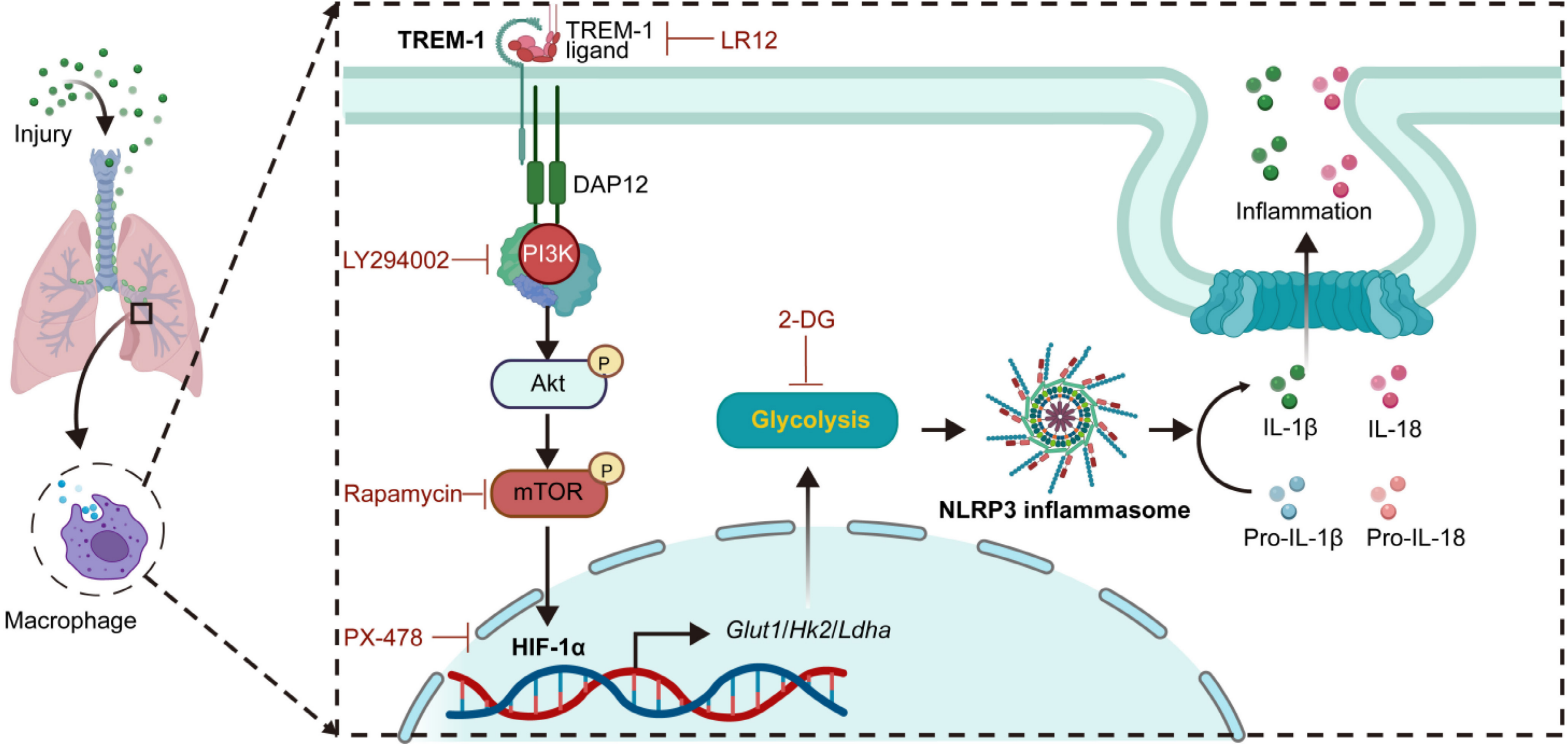
Schematic illustration. TREM-1 activation stimulates HIF-1α induced glucose metabolic reprogramming *via* PI3K/AKT/mTOR pathway. HIF-1α-induced glycolysis promotes TREM-1-governed NLRP3 inflammasome activation, facilitating intrapulmonary inflammation in ALI.

In this study, we found that TREM-1 activation induced a glucose metabolic reprogramming of macrophages *via* mTOR/HIF-1α signaling, which triggered NLRP3 inflammasome activation in ALI (**Figure 9**). Our previous study found blocking TREM-1 partially inhibits NLRP3 inflammasome activation in ALI. Then in this study, we observed that blocking TREM-1 also limited glycolysis in ALI mice. *In vitro*, TREM-1 activation orchestrated macrophage metabolic modifications towards increased glycolytic activity and decreased OXPHOS. And using glycolysis inhibition partially inhibited TREM-1-induced NLRP3 inflammasome activation in macrophages. HIF-1α, a critical transcriptional regulator of glycolysis, was induced by TREM-1 activation via the PI3K/AKT/mTOR pathway. Inhibition of mTOR or HIF-1α also prevented TREM-1-governed NLRP3 inflammasome activation in macrophages. Overall, the mTOR/HIF-1α/glycolysis pathway is a novel mechanism underlying TREM-1-governed NLRP3 inflammasome activation.

The crosstalk between TREM-1 and NLRP3 inflammasome has emerged as a novel mechanism of the inflammatory cascade in ALI. We have reported that TREM-1 blockade with LR12 inhibits the NLRP3 inflammasome activation in ALI 8. Others have shown that TREM-1 inhibition with synthetic peptide LP17 ameliorates neuroinflammatory injury and chronic obstructive pulmonary disease (COPD) *via* NLRP3 inflammasome-mediated pyroptosis 37, 38. TREM-1 blockade with LP17 restrains NLRP3/caspase-1 activation through SYK in microglia 39. Recent studies have shown that TREM-1 serves as a receptor for extracellular cold-inducible RNA-binding protein (eCIRP) to induce inflammation in ALI 40. Previous studies have been performed with TREM-1 inhibitory peptides, which was an indirect effect. Here, we first demonstrate that TREM-1 signaling using an agonist anti-TREM-1 Ab (Mab1187) promoted the priming and activation of NLRP3 inflammasome in macrophages. Our recent studies identified NLRP3 inflammasome as a new trigger of TREM-1 signaling. HMGB1 and IL-18 released by NLRP3 inflammasome triggered the TREM-1-amplified response 41. Those findings suggest that TREM-1 and NLRP3 inflammasome forms a positive feedback loop, promoting pulmonary inflammation.

Our study clarified the induction mechanism of glucose metabolism reprogramming during ALI. We have reported that glycolysis is a deteriorative factor in ALI 16. While its induction mechanism has not been thoroughly elucidated. Herein, our findings identify TREM-1 as a glycolytic stimulus, as evidenced by accentuated glucose consumption and lactate secretion and up-regulation of glycolytic metabolic enzymes in macrophages. Blocking TREM-1 attenuated intrapulmonary inflammation and limited glycolysis. Moreover, TREM-1 was found to promote mitochondrial fission in macrophages by facilitating DRP1^s616^ phosphorylation. Mitochondrial dynamic controls macrophages' fate through metabolic programming 42. Fission in macrophages leads to cristae expansion, reducing electron transport chain (ETC) efficiency and decreasing OXPHOS 43. These results are in line with an observation from IL-34, where IL-34 reprograms naïve myeloid cells into glycolytic macrophages 44. Also reported, most microbial stimuli increase glycolysis, but only stimulating the TLR4 with LPS leads to an increase in glycolysis. Instead, stimulation of TLR2 increases oxygen consumption and mitochondrial enzyme activity in monocytes 45. Furthermore, we found that TREM-1 instigates metabolic reprogramming in a PI3K/AKT/HIF-1α-dependent manner. Interestingly, ROS scavenger did not influence TREM-1 activated-HIF-1α. This observation is in line with the observation that mitochondrial, cytosolic, or lipid ROS were unnecessary for HIF-1α stability and transcription 46.

Glucose metabolites are thought to regulate the activation of the NLRP3 inflammasome 13. Here, we found that TREM-1 triggers NLRP3/caspase-1 activation and promotes IL-1β secretion. Using glycolysis inhibitor 2-DG reduced TREM-1 activation induced-NLRP3 inflammasomes. Glycolysis not only provides energy but also intermediates to work as a signal molecule activating NLRP3 inflammasome, e.g., Up-regulation of HK1-dependent glycolysis by mTOR regulates NLRP3 inflammasome activation 15. PKM2-dependent glycolysis promotes NLRP3 inflammasome activation by modulating the eukaryotic translation initiation factor 2 alpha kinase 2 phosphorylation in macrophages 47. Lactate is essential for NLRP3 inflammasome activation 48. Our results showed that inhibition of glycolysis with 2‐DG suppresses TREM-1 protein in mice with ALI induced by LPS. Our previous studies showed that inflammatory response precedes enhanced glycolysis during the development of ALI, and inflammation could induce glycolysis 16. Glycolysis and its intermediate metabolites are involved in inflammation as signaling molecules. In addition, Glycolysis, potentially through reactive aldehydes and a redox-dependent mechanism, exerts positive feedback on the inflammatory transcription factors 49. Intermediate metabolites of glycolysis can promote the expression of TREM-1 ligands and inducers. For example, PKM2, a key enzyme in glycolysis, regulates the Warburg effect and promotes HMGB1 release 47, 50. HMGB1 has been suggested as a TREM-1 ligand 51. PKM2 promotes cyclooxygenase (COX)-2 52 and HIF-1α-dependent transcriptional up-regulation of COX-2, which regulates the expression of TREM-1 53. This result suggests that inhibiting glycolysis reduced the expression of the TREM-1 ligand or inducer by inhibiting glycolysis. HIF-1α, a key transcription factor in glycolysis, mediates NLRP3 inflammasome activation in synovial fibrosis 54. PI3K inhibitor attenuates NLRP3 inflammasome activation in neural stem cells 55. Our results also found inhibition of the mTOR signal or HIF-1α reduced TREM-1-induced NLRP3 inflammasome activation. Collectively, the findings indicate that TREM-1 triggers NLRP3 inflammasome in an mTOR/HIF-1α/glycolysis-dependent manner.

Our preclinical data are promising in the therapeutic potential of TREM-1, while its downstream metabolic intermediates remain to be elucidated. Further studies are needed to understand how glycolysis participates in TREM-1-induced NLRP3 inflammasome. N-acetylglucosamine reportedly promotes untethering of HK2 from the mitochondria, which is sufficient to drive NLRP3 inflammasome activation 56. The mechanisms of mTOR, HK2, and HIF-1α involved in TREM-1-triggered NLRP3 inflammasome activation require further exploration.

In conclusion, our study reveals a crucial role of TREM-1 in controlling glucose metabolism *via* the HIF-1α pathway. This glucose metabolic reprogramming by TREM-1 is vital to the NLRP3 inflammasome activation. The mTOR/HIF-1α/glycolysis pathway in macrophages may thus be a novel strategy for the treatment of TREM-1- and NLRP3 inflammasome-associated inflammatory diseases.

## Supplementary Material

Supplementary figures.

## Figures and Tables

**Table 1:** Antibody sources and dilutions

antibody	source	catalog	Dilution ratio
Anti-IL-1β polyclonal antibody	R&D	AF-401-NA	1:2000
Anti-iNOS polyclonal antibody	Proteintech	18985-1-AP	1:2000
Anti-HK2 monoclonal antibody	CST	#2867	1:2000
Anti-mTOR monoclonal antibody	Proteintech	66888-1-lg	1:2000
Anti-phospho-mTOR (Ser2448) monoclonal Antibody	Proteintech	67778-1-lg	1:2000
Anti-HIF-1α-monoclonal antibody	Novus	NB100-105	1:1500
Anti-LDHA-monoclonal antibody	Abcam	Ab52488	1:10000
Anti-NLRP3 monoclonal antibody	CST	#12721	1:2000
Anti-procaspase1/p10/p20 monoclonal antibody	Abcam	Ab179515	1:1000
Anti-total OXPHOS complexes antibody	Abcam	Ab110413	1:1000
Anti-Phospho-PI3K p85 alpha (Tyr607) -polyclonal antibody	Affinity	AF3241	1:1000
Anti-pan-AKT1/2/3-polyclonal antibody	Affinity	AF6261	1:1000
Anti-pan-Phospho-pan-AKT1/2/3 (Ser473) -polyclonal antibody	Affinity	AF0016	1:1000
Anti-β-actin polyclonal antibody	SAB	#21338	1:7500
Anti-α-tubulin monoclonal antibody	Servicebio	GB11200	1:10000
Anti-GAPDH monoclonal antibody	Servicebio	GB11002	1:2000

**Table 2:** Sequences of the primers used in this study

Gene	Forward primer (5'-3')	Reverse primer (5'-3')
*Glut1*	CAGTTCGGCTATAACACTGGTG	GCCCCCGACAGAGAAGATG
*Hk2*	TGATCGCCTGCTTATTCACGG	AACCGCCTAGAAATCTCCAGA
*Pkm2*	GCCGCCTGGACATTGACTC	CCATGAGAGAAATTCAGCCGAG
*Pfkfb3*	CAACTCCCCAACCGTGATTGT	GAGGTAGCGAGTCAGCTTCTT
*Ldha*	TGTCTCCAGCAAAGACTACTGT	GACTGTACTTGACAATGTTGGGA
*Hif-1α*	ACCTTCATCGGAAACTCCAAAG	ACTGTTAGGCTCAGGTGAACT
*Nlrp3*	TACGGCCGTCTACGTCTTCT	CGCAGATCACACTCCTCAAA
*pro-caspase-1β*	CACAGCTCTGGAGATGGTGA	CTTTCAAGCTTGGGCACTTC
*pro-Il-1β*	CAGGCAGGCAGTATCACTCA	AGCTCATATGGGTCCGACAG
*Tnf-α*	AGCCCCCAGTCTGTATCCTT	CTCCCTTTGCAGAACTCAGG
*Trem-1*	CTGTGCGTGTTCTTTGTC	CTTCCCGTCTGGTAGTCT
*β-actin*	TTCCAGCCTTCCTTCTTG	GGAGCCAGAGCA GTAATC

## Abbreviations

ALI: acute lung injury
ATP5A: ATP synthase subunit alpha
BSA: albumin from bovine serum
COPD: obstructive pulmonary disease
DAP12: adaptor protein 12-kDa DNAX activating protein
DRP1: dynamin-related protein 1
eCIRP: extracellular cold-inducible RNA-binding protein
eIF-4E-BP1: 4E-binding protein-1
ETC: electron transport chain
GLUT1: glucose transporter type 1
HIF-1α: hypoxia-inducible factor-1α
HK2: hexokinase2
HMGB1: high-mobility group protein B1
H&E: haematoxylin and eosin
LPS: lipopolysaccharide
iNOS: nitric oxide synthase
IL-1β: interleukin-1 beta
LDHA: lactate dehydrogenase A
TLR: Toll-like receptor
mTOR: mammalian target of Rapamycin
MTCO1: cytochrome c oxidase subunit 1
NLRP3: NOD-, LRR- and pyrin domain-containing 3
NDUFB8: NADH dehydrogenase beta subcomplex subunit 8
NAC: N-Acetylcysteine
OXPHOS: oxidetive phosphorylation
PHDs: prolyl hydroxylases
PI3K: phosphoinositide 3-kinase
PKM2: pyruvate dehydrogenase kinase M2
ROS: reactive oxygen species
SDHB: succinate dehydrogenase subunit B
TREM-1: triggering receptor expressed on myeloid cells-1
TNF-α: tumor necrosis factor-alpha
UQCRC2: cytochrome b-c1 complex subunit 2
