# Supplementary material for: TREM-1 governs NLRP3 inflammasome activation of macrophages by firing up glycolysis in acute lung injury
Source: Int J Biol Sci. 2023 Jan 1;19(1):242–57. doi: 10.7150/ijbs.77304 (PMC9760435; doi:10.7150/ijbs.77304)
Supplement: Supplementary file 1 — Supplementary figures. [file ijbsv19p0242s1.pdf]

Supplementary material

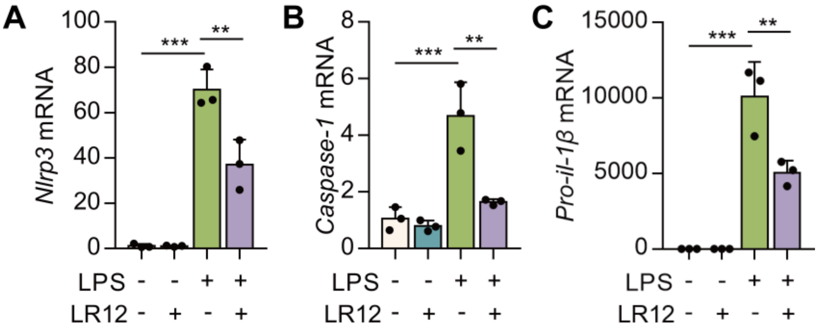

Supplementary Figure 1. LR12 pretreatment attenuates the expressions of *Nlrp3*, *Pro-caspase-1*, and *Pro-il-1β* mRNA in LPS-induced macrophages. Macrophages were treated with LR12 (25 µg/mL) 30 min before LPS (100 ng/mL) stimulation. **(A-C)** Six hours later, the mRNA level of *Nlrp3, Pro-caspase-1, and Pro-il-1β* was detected by real-time PCR. Data are expressed as the mean ± SD. One-way ANOVA adjusted by Tukey's multiple comparison test was used. ** $P < 0.01$ and *** $P < 0.001$.

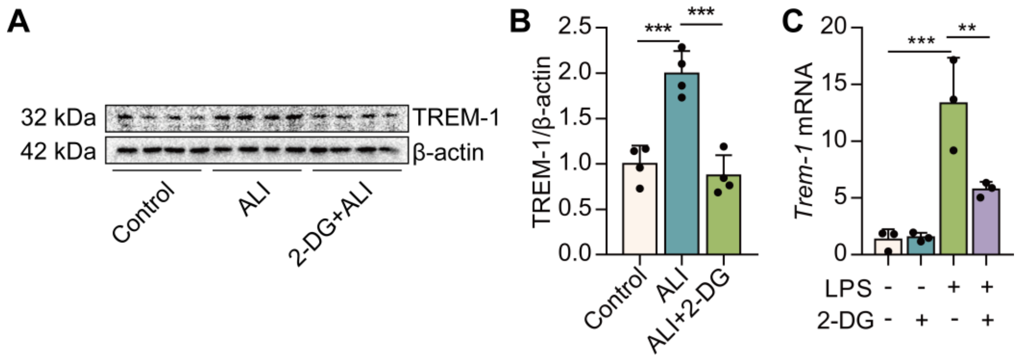

Supplementary Figure 2. 2-DG pretreatment attenuates the expressions of TREM-1 expression in the lung of LPS-treated mice or LPS-induced macrophages. C57BL/6J mice received LPS (5 mg/kg, *it.*) with or without 2-DG pretreatment (1 g/kg) for 1 h. **(A-B)** The expression of TREM-1 protein (*n*=4) in the lung 12 h after the LPS injection was detected by western blot. **(C)** Macrophages were treated by LPS (100 ng/ml) with or without 2-DG pretreatment (5 mM). The expression of *Trem-1* mRNA in macrophages 6 h after the LPS administration was detected by real-time PCR. Data are expressed as the mean ± SD. One-way ANOVA adjusted by Tukey's multiple comparison test was used. ** $P < 0.01$ and *** $P < 0.001$.

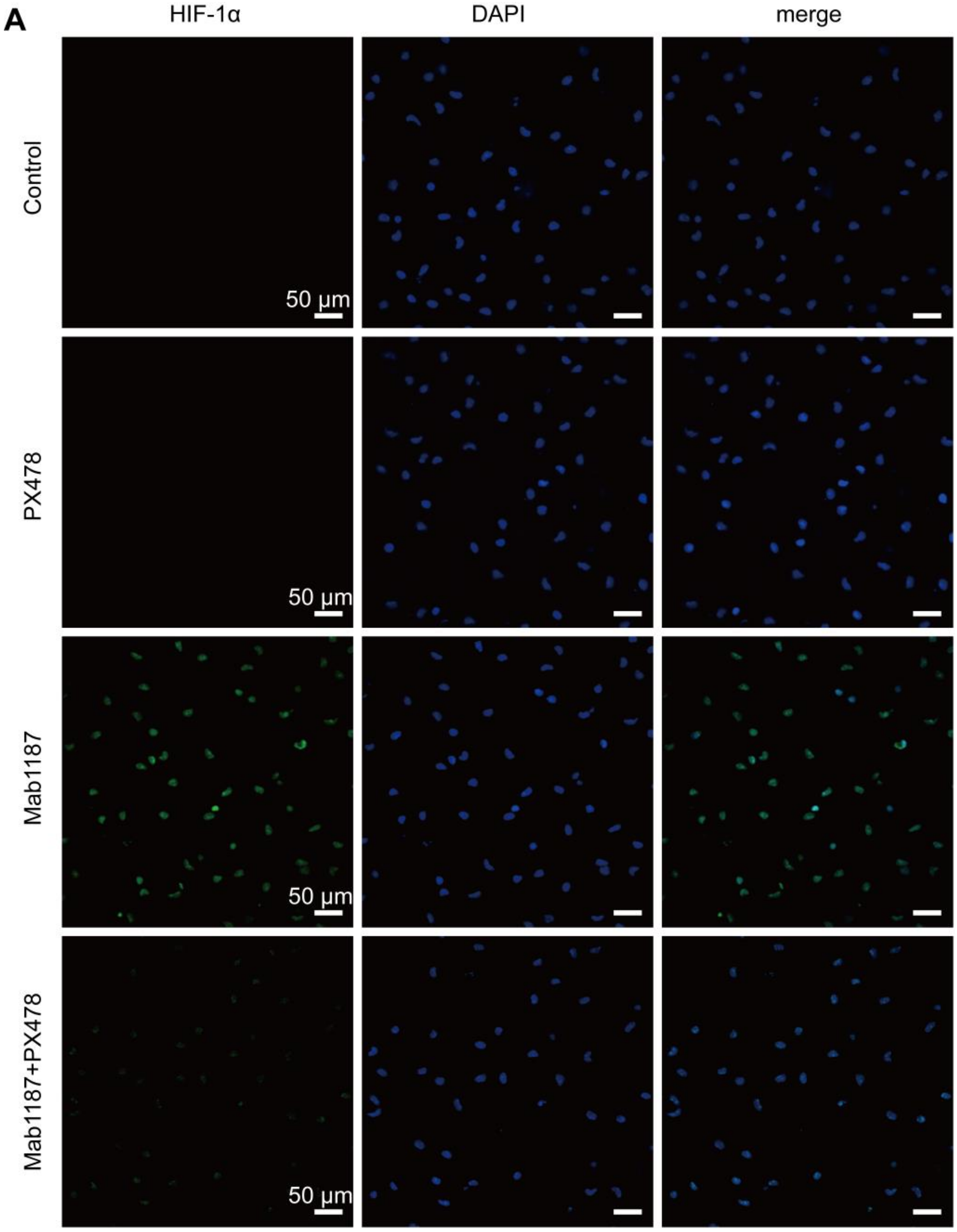

Supplementary Figure 3. PX-478 significantly inhibited HIF-1α accumulation and translocation to the nucleus induced by TREM-1 activation. Macrophages (2×10^5 cells/well) were premixed with PBS control or PX-478 (25 μM) for 30 min, then plated into 24-well plates with agonist anti-TREM-1 mAb (10 μg/mL) for 24 h. **(A)** Macrophages were subjected to immunofluorescence examination to analyze the HIF-1α accumulation and translocation to the nucleus (scale bar, 50 μm).

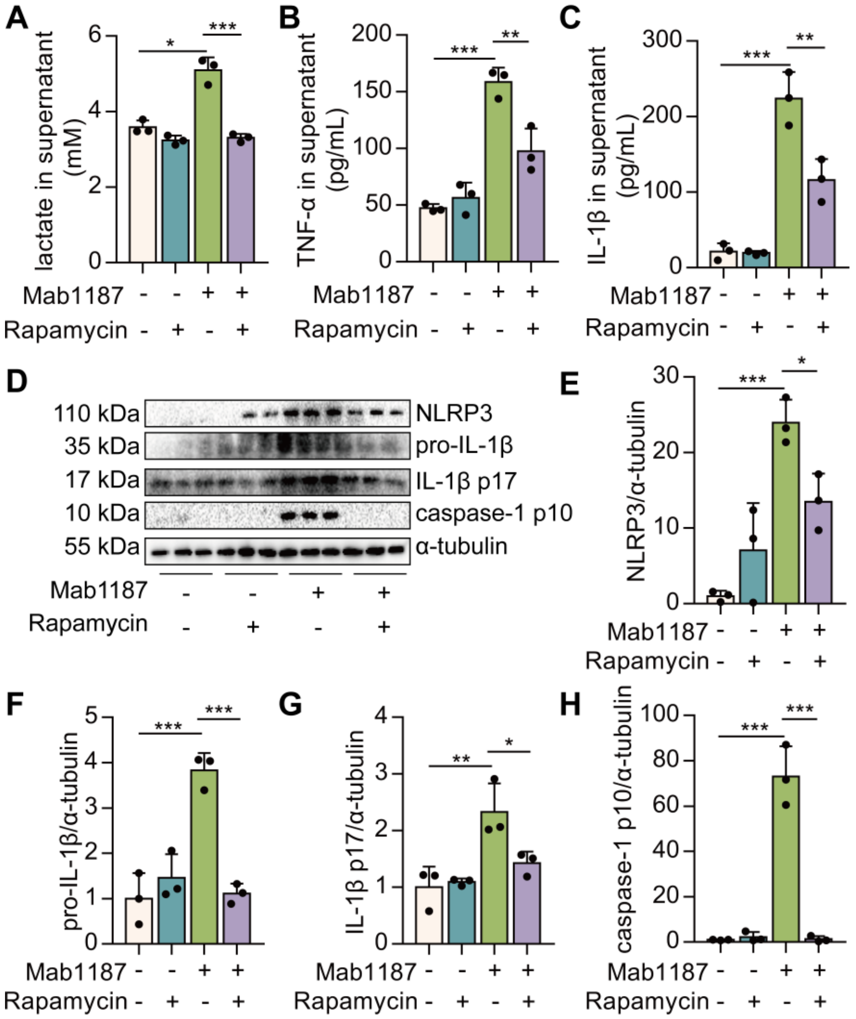

Supplementary Figure 4. TREM-1-mediated NLRP3 inflammasome activation depends on mTOR. **(A)** 1×10^6 macrophages/well were premixed with PBS control or rapamycin (100 nM) and then plated into 12-well plates with agonist anti-TREM-1 mAb (10 µg/mL). After 24 h, supernatants were analyzed for lactate production, *n*=3. **(B-C)** TNF-α and IL-1β production in the supernatant was measured by ELISA, *n*=3. **(D)** NLRP3, pro-IL-1β, IL-1β p17, and caspase-1 p10 protein in cell lysate were detected by western blot, *n*=3. **(E-H)** Quantification of indicated protein levels in (D), *n*=3. *n*=3 biological replicates. Data are expressed as the mean ± SD. One-way ANOVA adjusted by Tukey's multiple comparison test was used. * $P < 0.05$, ** $P < 0.01$, and *** $P < 0.001$.

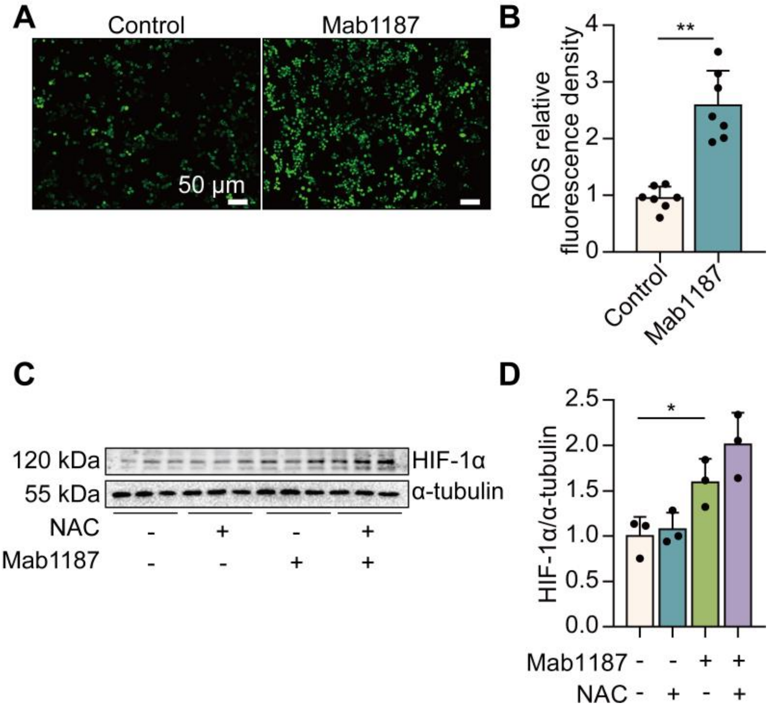

Supplementary Figure 5. TREM-1-mediated HIF-1α accumulation is independent of ROS. **(A)** Macrophages were plated in 24-well plates with agonist anti-TREM-1 mAb (10 μg/mL). After 24 h, intracellular ROS was measured (scale bar, 50 μm). **(B)** Average fluorescent intensity was calculated by Image J, *n*=7. **(C)** 1×10^6 macrophages/well were premixed with PBS control or NAC (500 μM) and then plated into 12-well plates with agonist anti-TREM-1 mAb (10 μg/mL). After 24 h, the protein of HIF-1α was performed by western blot with α-tubulin as a loading control, *n*=3. **(D)** Quantification of indicated protein levels in (C), *n*=3. *n*=3 biological replicates. Data are expressed as the mean ± SD. The student's *t*-test (two-tailed, unpaired) was used to compare Mab1187 and Control in B: ** $P < 0.01$. One-way ANOVA adjusted by Tukey's multiple comparison test was used in D: * $P < 0.05$.
